# Estimated Modern Use: Employing A Service Statistics-Based Indicator to Monitor Family Planning Programs

**DOI:** 10.12688/gatesopenres.16346.1

**Published:** 2025-05-07

**Authors:** Kristin Bietsch, Margaret Reeves, Jessica Williamson, Priya Emmart, Emily Sonneveldt

**Affiliations:** 1 Avenir Health, Glastonbury, Connecticut, USA

**Keywords:** Estimated Modern Use (EMU), family planning, contraceptive use indicator, routine service statistics, program monitoring, HMIS

## Abstract

Estimated Modern Use (EMU) is a novel, service statistics-based indicator designed to complement Couple Years of Protection (CYP) in assessing the scale of family planning use and the first widely used metric since CYPs. Developed by the Track20 project, EMU offers a population-based proportional metric that facilitates cross-country comparisons and temporal trend analysis. By leveraging existing family planning service statistics, EMU provides a more accessible and interpretable measure of contraceptive use.

The associated SS-to-EMU tool used to calculate EMU incorporates rigorous data quality review mechanisms, including data visualizations and validated review processes, to enhance the reliability and utility of family planning data for decision-making. The standardization of EMU across countries and projects promotes its integration into routine data review practices, fostering a more comprehensive approach to family planning monitoring and evaluation. Since 2014, all countries that prepare annual estimates for the FP2030 global initiative utilize the SS to EMU tool, to assess data quality and produce EMU estimates.

Moreover, the EMU serves as a valuable input for the Family Planning Estimation Tool (FPET), contributing to the refinement of modeled estimates of modern contraceptive prevalence. Since its introduction, EMU has gained widespread adoption at various levels, demonstrating its effectiveness in informing global, regional, and country-level monitoring efforts. Ongoing refinements to the EMU calculation further enhance its accuracy and utility as a supplementary data source for understanding contraceptive use patterns.

## Background

The global family planning landscape has witnessed a paradigm shift since the launch of FP2020 in 2012. The Track20 project [ 1], which supports the measurement mandate of FP2020, now FP2030, sought to establish a standardized approach for monitoring progress across countries. Historically, family planning programs have relied on two primary data sources: survey data and routine service statistics.

Survey data, such as those collected through Demographic and Health Surveys [ 2] (DHS) and Multiple Indicator Cluster Surveys [ 3] (MICS), provide snapshots of contraceptive use at specific points in time. However, lags between surveys limit their utility for real-time monitoring and decision-making. In contrast, routine service statistics, gathered through Health Management Information Systems (HMIS) and Logistic Management Information Systems (LMIS), offer a more frequent but often less comprehensive view of program activities. Countries rely on their routine data to make decisions about resource allocation, distribution and procurement of commodities, all of which have important budgetary and spending implications. Differences in the data elements and methodologies used by countries to calculate national coverage indicators make cross-country comparison difficult, and while CYPs are widely referenced, the concept is not well suited as a measure of coverage.

To address the limitations of these traditional data sources, Track20 sought to create a standardized approach to transform routine service statistics into a single metric that could track progress between surveys and could contribute to more frequent estimations of contraceptive prevalence through modeling. This led to the development of the Estimated Modern Use (EMU) indicator and its integration into the Family Planning Estimation Tool (FPET). ^1^^–^^4^

EMU offers a valuable addition to the family planning monitoring toolkit. By providing a standardized measure of contraceptive coverage that can be calculated at different administrative levels using consistently available data, EMU facilitates real-time monitoring and enables more informed decision-making. It also provides a standardized way to measure the volume of use from service statistics that can help minimize confusion across countries’ differing approaches. While EMU may not perfectly mirror levels of contraceptive use, its effectiveness has been demonstrated in tracking trends over time, making it a valuable complement to survey data. ^5^^,^^6^

EMU serves as a standalone indicator that can be used to assess program performance, identify areas for improvement, and inform resource allocation decisions. The EMU’s standardized methodology and flexibility in accommodating diverse data types ensures its comparability across countries, making it a valuable tool for global family planning monitoring and evaluation. It also solves for an important prior constraint in family planning metrics, the desire for an estimate of the number of users. The number of countries that directly produce users through their HMIS is very small.

## Methods

### Calculating Estimated Modern Use

The EMU calculation is the number of estimated modern users divided by the population of women of reproductive age (WRA). The numerator combines service statistics by method with CYP factors (how long a particular method will provide protection against pregnancy) and continuation rates derived from CYP factors for long acting methods.

$$\mathrm{EMU}=\frac{\text{Estimated Modern Users}}{\text{Population}\;\left(\mathrm{WRA}\right)}$$

Where:

Estimated Modern Users are Family Planning (FP) Service Statistics (by method) using CYP factors and long acting permanent method (LAPM) continuation rates.

Population (WRA) is the population of women of reproductive age (15-49), representing the pool of potential contraceptive users.

As metrics, EMU and CYP start from a similar calculation, which is designed to estimate an aggregated impact from a variety of contraceptive commodities on providing protection against unintended pregnancy. CYPs are calculated as a direct conversion of contraceptive commodities (ex. 10 IUDs) into an indicator of the impact of those commodities (46 Couple Years of Protection, all allocated to the year of insertion). The CYP also serves as a single indicator representing the combined impact of different contraceptive methods with different levels of effectiveness and duration. The EMU annualizes the CYP data – carrying users over for the duration of their method while accounting for discontinuation before dividing by population. By producing a proportional indicator, rather than an absolute number, the EMU is more comparable to mCP and better aligns with annual monitoring and comparisons between regions and countries.

To account for system and data variations and better represent levels of FP use over time, the EMU calculation makes several adjustments to the raw data during the process of converting routine service statistics to FP Users for the EMU numerator. Details on the conversion to FP Users and other adjustments are provided in the following sections.

#### Estimating FP Users

Except for a few countries that collect true FP Users data, which tracks individual users over time, FP routine service statistics data in most countries do not represent actual FP use, but rather, a count of FP services provided, or FP commodities distributed. To account for this, some adjustments are made to the raw data provided on FP Commodities or FP Visits to convert them to estimated FP Users to produce the numerator for the EMU. The adjustments made to convert the raw service statistics into FP Users considers 3 groups of FP Users in any given year:

1. Current Users: women using a method in the same year it was provided.
2. Continuing Users: women still using a long acting or permanent method (LAPM) provided in a prior year for which data was available.
3. Historic Users: women we assume are still using a LAPM provided prior to the first year of data available.

***Current Users***

To estimate the number of current FP Users in the year a service was provided, we vary our assumptions by method type.

For short-term methods, those methods that are only effective for a short duration and require refills or revisits within the same year to continue to protect against pregnancy, we use the globally accepted CYP factors to convert services or products into users. For example, four visits for DMPA-IM, or 4 injectables dispensed, is equal to one CYP and one estimated modern user.

Using condom distribution numbers from HMIS is particularly challenging. It can be difficult to determine the number of condoms used specifically to prevent pregnancy because they may be distributed for multiple purposes and through non-FP programs and are often left for clients to take freely instead of distributed in a carefully documented manner. Because of this, in countries where condoms are not a common method of family planning, they may be excluded from EMU calculations.

**Short-Term Methods**

$$U_{m_{\mathrm{STM}},s,t}=C_{m_{\mathrm{STM}},s,t}\times \frac{1}{{\mathrm{CYP}}_{m_{\mathrm{STM}},s}}$$

Where:

$m_{\mathrm{STM}}$ is the type of short-term method,

*s* is the type of service statistic (commodities to clients, commodities to facilities, visits),

*t* is year,

$U_{m_{\mathrm{STM}},s,t}$ is the number of users of a short-term method, $m_{\mathrm{STM}}$ , of service statistics type *s,* in year *t,*

$C_{m_{\mathrm{STM}},s,t}\;$ is the number of commodities or visits of the short-term method, $m_{\mathrm{STM}}$ , of service statistics type *s,* in year *t*

${\mathrm{CYP}}_{m_{\mathrm{STM}},s}\;$ is the couple years protection for the short-term method, $m_{\mathrm{STM}}$ , and service statistics type, *s*

For long acting reversible methods, which are provided in a given year but can continue to provide protection in subsequent years, we estimate that each service or product (IUD/Implant) inserted is equivalent to one user, with some small adjustment to account for first-year discontinuation (for example, CYP continuation curves estimate there is a 92% continuation in the first year for Copper T IUDs). More discussion on how the calculation accounts for future years is included in the next section on “Continuing Users.”

**Long-Term Method, Current Users**

$$U_{m_{\mathrm{LTM}},s,t}^{\mathrm{Current}}=C_{m_{\mathrm{LTM}},s,t}\times {\mathrm{Con}}_{m_{\mathrm{LTM}},1}$$

Where:

$m_{\mathrm{LTM}}$ is the type of long-term method,

*s* is the type of service statistic (commodities to clients, commodities to facilities, visits),

*t* is year,

$U_{m_{\mathrm{LTM}},s,t}^{\mathrm{Current}}$ is the number of current users (who received their method in year *t*) of a long-term method, $m_{\mathrm{LTM}}$ , of service statistics type *s,* in year *t,*

$C_{m_{\mathrm{LTM}},s,t}\;$ is the number of commodities or visits of the long-term method, $m_{\mathrm{LTM}}$ , of service statistics type *s,* in year *t*

${\mathrm{Con}}_{m_{\mathrm{LTM}},1}$ is the continuation rate of method $m_{\mathrm{LTM}}$ in year 1

For Permanent Methods, which are provided in a given year but continue to provide protection for the duration of the user’s reproductive life, we estimate that each service is equal to one user in that year. More discussion on how the calculation accounts for future years is included in the next section on “Continuing Users”.

***Continuing Users***

**Figure 1.**
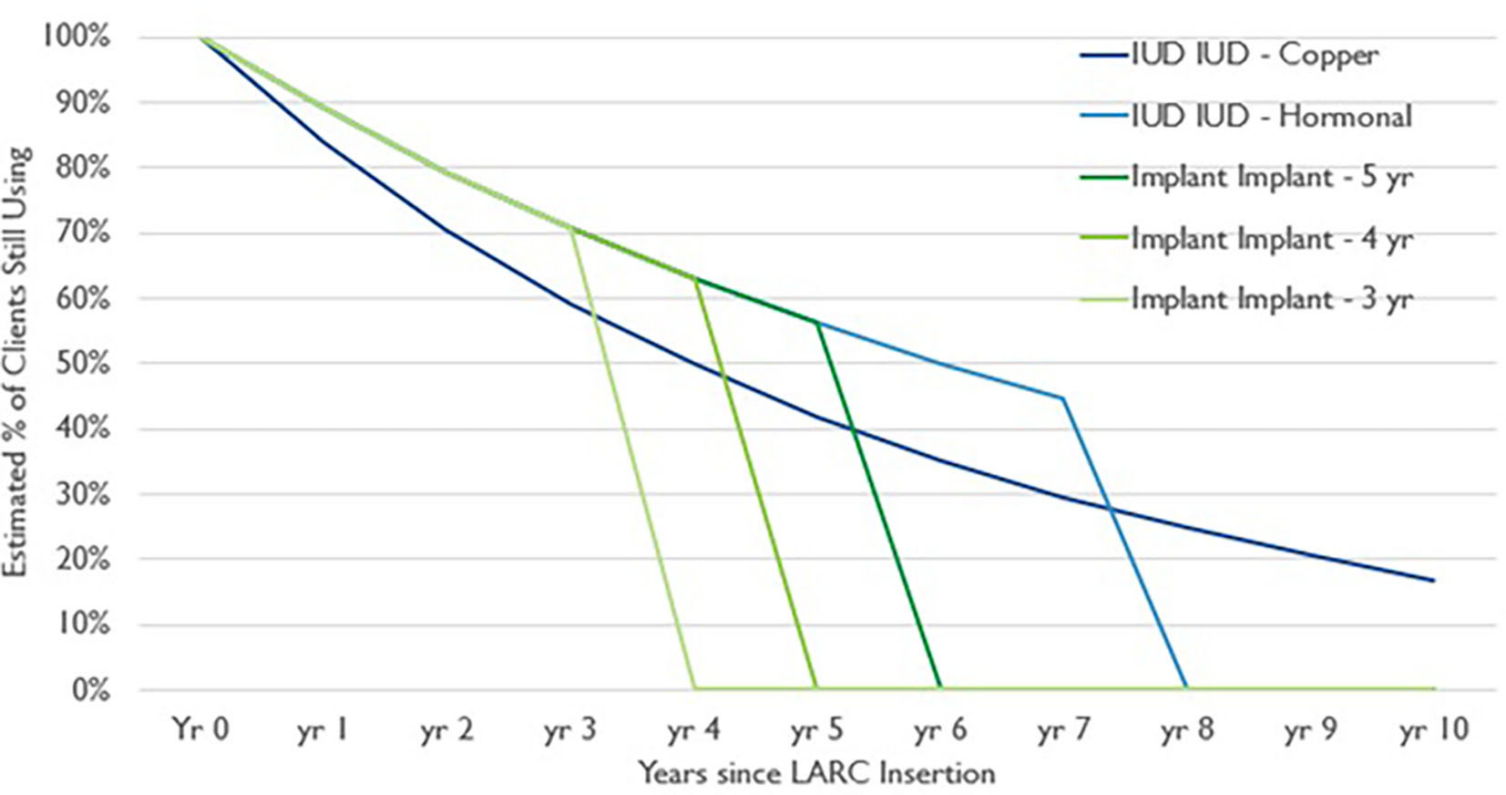
Long acting reversible contraception continuation curves.

A critical difference between CYP and EMU is the distribution of contraceptive use over time. CYPs apply the full impact of long acting and permanent methods (LAPMs) to the year in which the method was provided. When counted in this way, an implant, for example, would show benefit in the year it was inserted, but no impact in future years, even though it continues to provide contraceptive protection for several years into the future. A direct conversion of CYPs to FP Users would result in an overestimation of users in the year of service provision and can skew trends in users when rapid scale-up or declines in service provision occur. In the EMU calculation, a “carryover” approach is used, in which that impact is distributed across the years a method would be in use, based on the standard continuation rates used to develop the CYP factors ^7^ ( Figure 1).

**Long-term Method, Continuing Users**

$$U_{m_{\mathrm{LTM}},s,t}^{\mathrm{Continuing}}=\sum_{x=1}^{t-t_{0}}\left(C_{m_{\mathrm{LTM}},s,t-x}\times {\mathrm{Con}}_{m_{\mathrm{LTM}},x+1}\right)$$

$m_{\mathrm{LTM}}$ is the type of long-term method,

*s* is the type of service statistic (commodities to clients, commodities to facilities, visits),

*t* is year,

$t_{0}$ is the first year of data collection,

$U_{m_{\mathrm{LTM}},s,t}^{\mathrm{Continuing}}$ is the number of continuing users (who received their method during data collection, prior to year *t*, and are still expected to be users in year *t*) of a long-term method, $m_{\mathrm{LTM}}$ , of service statistics type *s,* in year *t,*

$C_{m_{\mathrm{LTM}},s,t-x}\;$ is the number of commodities or visits of the long-term method, $m_{\mathrm{LTM}}$ , of service statistics type *s, x* years prior to year *t*

${\mathrm{Con}}_{m_{\mathrm{LTM}},x+1}$ is the continuation rate of method $m_{\mathrm{LTM}}$ in *x*+1 years of use

**Figure 2.**
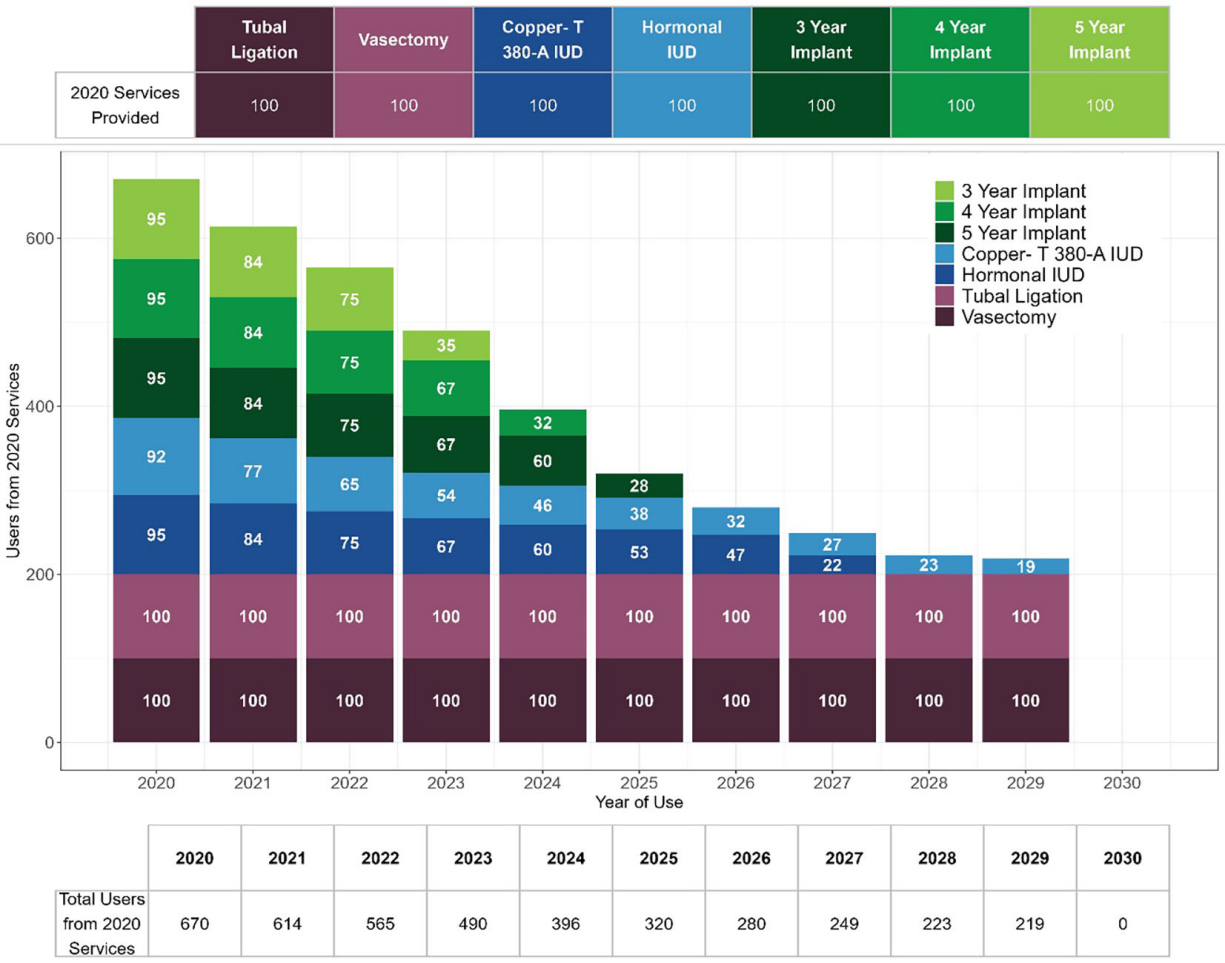
Estimated users for long acting and permanent methods from one year of services.

In the example illustrated in Figure 2, 100 of each LAPM were provided in 2020. Based on those services in 2020, we estimate 670 users in 2020. Each year some of those users are assumed to continue using their method until it is removed, no longer effective, or until they age out of “childbearing age”. By 2030, we assume all of those users who initiated in 2020 are no longer using that method, or in the case of sterilization, no longer of childbearing age. As a specific example, if a Copper-T 10-year IUD was inserted in 2020, all 4.6 CYPs would be counted in that year. However, because EMU estimates users in a given year, if 100 Cooper-T are inserted in 2020, the SS-to-EMU tool assumes that 92 will be used for a year, 77 for two years, 65 for three years and so on, following the continuation curve which underlies the CYP calculation. This serves to smooth out trends in long acting contraceptive use, as there may be fluctuations in the acceptance of LAPMs by clients which are unlikely to result in immediate fluctuations in use as women continue to use these methods over several years.

One limitation of the current approach is that a single global continuation curve is used for each method, which does not take into account the country-specific variability in method continuation indicated by DHS data. Limitations to a country-specific approach include lack of discontinuation data for many countries and timeliness of data for newly popular methods when using a historical reproductive calendar for calculating discontinuation rates. The continuation rates from CYP estimates are calculated using results from multiple studies. Cohort studies were prioritized for the CYP analysis, to reduce recall bias from the retrospective calendars in DHS. However, there were not enough cohort studies to rely solely on this approach, so the estimates were created using a combination of cohort data and secondary analysis of DHS data.

Track20 tested using regional discontinuation rates, but the variation within regions (among countries with discontinuation data) were the same in scale as those using the global rates, meaning that they did not improve on the calculation. Future testing could include different ways to reflect country variation.

***Historic Users***

As previously discussed, LAPM services require special consideration when estimating users because their use/impact can continue for up to 10 years [ 4] after a service is provided. Routine service statistics data are often limited in terms of the time trends available due to system changes and lack of electronification of past service data. As a result, it is common that countries have less than 10 years of service statistics data available in their current system, meaning that there are women using LAPMs that were provided before the first year of available data, but about whom we have no information. Disregarding these users runs the risk of substantially underestimating current levels of contraceptive use, especially in countries where a greater proportion of women use LAPMs, as it would assume that no service provision occurred before the first year of available data.

**Figure 3.**
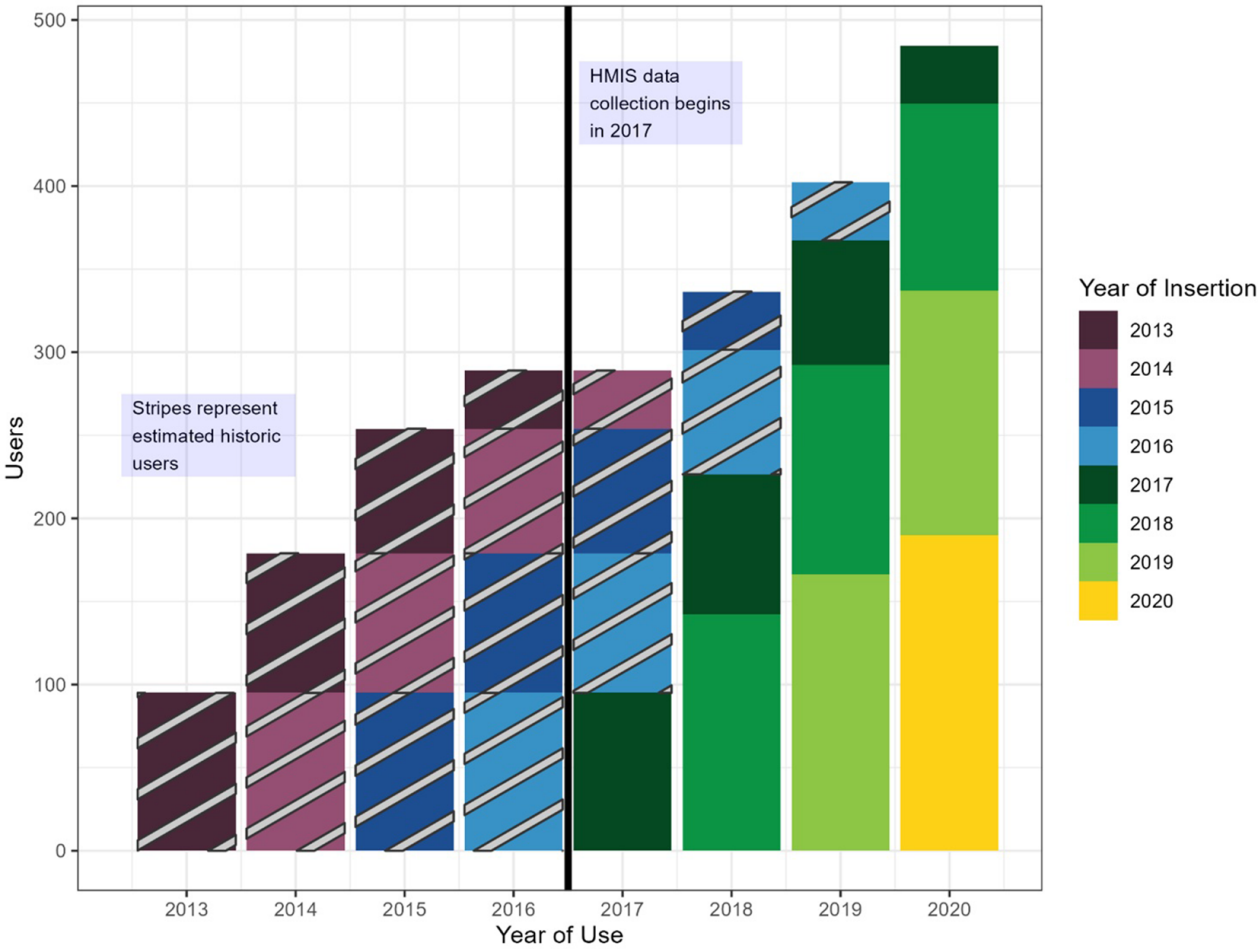
Estimated implanon users, by year of use and year of insertion.

To estimate “historic users” (those still using a method provided before the first year of available data), we make some assumptions about LAPM distribution before the first year of available data. The calculation of historic users is based upon the maturity of the FP program in the first year of data collection for each LAPM- was provision of this method already at scale, still scaling up, or just beginning the year data collection began? If provision of the method started in the first year of data collection, we assume there are no historic users. If the method being provided was at scale in the first year of data collection, we assume that the number of insertions in previous years was similar to the number of insertions in the first year of data collection. If the program was scaling up distribution of this method, we assume the number of historical users falls between the two other scenarios. For example, if a data system began recording methods distributed in 2017, but the country began inserting Implanon in 2013, we expect that some women received implants in 2016 or earlier and are still implant users in 2017 and beyond ( Figure 3). Based on the scenario identified and the first year of data available, the EMU calculation estimates the number of women who received LAPMs before the first year of data and then applies the same continuation trends used to estimate continuing users. This allows the EMU to better approximate actual contraceptive use, integrating LAPM users from prior years into the years for which data is available.

**Long-term Method, Historic Users**

$$U_{m_{\mathrm{LTM}},s,t}^{\mathrm{Historic}}=\left\{ \begin{array}{c} \mathrm{No}\;\mathrm{Historic}=0\\ \mathrm{Scaling}\;\mathrm{Up}=\frac{1}{2}\times C_{m_{\mathrm{LTM}},s,t_{0}}\times \sum_{x=t-t_{0}}^{L_{m_{\mathrm{LTM}}}^{\max}}{\mathrm{Con}}_{m_{\mathrm{LTM}},x+2}\\ \mathrm{At}\;\mathrm{Scale}=C_{m_{\mathrm{LTM}},s,t_{0}}\times \sum_{x=t-t_{0}}^{L_{m_{\mathrm{LTM}}}^{\max}}{\mathrm{Con}}_{m_{\mathrm{LTM}},x+2} \end{array}\right.$$

$m_{\mathrm{LTM}}$ is the type of long-term method,

*s* is the type of service statistic (commodities to clients, commodities to facilities, visits),

*t* is year,

$t_{0}$ is the first year of data collection,

$U_{m_{\mathrm{LTM}},s,t}^{\mathrm{Historic}}$ is the number of estimated historic users (who received their method before data collection started and are still expected to be users in year *t*) of a long-term method, $m_{\mathrm{LTM}}$ , of service statistics type *s,* in year *t,*

$C_{m_{\mathrm{LTM}},s,t_{0}}$ is the number of commodities or visits of the long-term method, $m_{\mathrm{LTM}}$ , of service statistics type *s,* in the first year of data collection, $t_{0}$

$L_{m_{\mathrm{LTM}}}^{\max}$ is the maximum number of years of continuation for long-term method, $m_{\mathrm{LTM}}$ ,

${\mathrm{Con}}_{m_{\mathrm{LTM}},x+2}$ is the continuation rate of method $m_{\mathrm{LTM}}$ in *x*+2 years of use

The extent to which these estimates impact a countries EMU depends on the country’s method mix, number of years they have data in their HMIS, and the extent to which their family planning program has changed or prioritized different methods in the recent past. A country that primarily relies on short acting methods will see little impact in their current number of users based on these estimations. Similarly, a country that has had a robust implant program for more than five years will not see an impact on estimates of current users, since the available data covers the period of effectiveness prior to the current year. These calculations will have the largest impact in countries that are scaling up long acting or permanent methods and have been doing so for only a few years. Importantly, as countries accumulate more years of data, these calculations have no impact on current data.

While historic users will always be an estimate, the benefits of this approach outweigh the risk of ignoring prior service provision, and those risks are minimized by our approach in the following ways:

1. This approach avoids the incorrect assumption that NO services were provided prior to the first year of data.
2. As countries have more data available, historic users have less impact on the total estimate. Once a country has 10 years’ worth of data available, historic users no longer factor into the EMU for the current year or future estimates.
3. Historic users’ presence in data is dependent on duration of method.
  a) “Historic” sterilization/IUDs last longest, but these methods generally represent a small proportion of use in most countries.
  b) “Historic” implants are present for a maximum of 5 years, depending on type of implant.
4. Adjustment for “new” or “scaling” versus “stable” trends helps to mitigate risk of over-estimating prior distribution.

Adding all three types of long-term method users together results in the estimated number of long-term methods users in a year.

**Long-term Methods, Total Users**

$$U_{m_{\mathrm{LTM}},s,t}=U_{m_{\mathrm{LTM}},s,t}^{\mathrm{Current}}+U_{m_{\mathrm{LTM}},s,t}^{\mathrm{Continuing}}+U_{m_{\mathrm{LTM}},s,t}^{\mathrm{Historic}}$$

Where:

$U_{m_{\mathrm{LTM}},s,t}$ is the total number of users of long-term method, $m_{\mathrm{LTM}}$ , of service statistics type *s,* in year *t,*

$U_{m_{\mathrm{LTM}},s,t}^{\mathrm{Current}}$ is the number of current users (who received their method in year *t*) of a long-term method, $m_{\mathrm{LTM}}$ , of service statistics type *s,* in year *t,*

$U_{m_{\mathrm{LTM}},s,t}^{\mathrm{Continuing}}$ is the number of continuing users (who received their method during data collection, prior to year *t*, and are still expected to be users in year *t*) of a long-term method, $m_{\mathrm{LTM}}$ , of service statistics type *s,* in year *t,*

$U_{m_{\mathrm{LTM}},s,t}^{\mathrm{Historic}}$ is the number of estimated historic users (who received their method before data collection started and are still expected to be users in year *t*) of a long-term method, $m_{\mathrm{LTM}}$ , of service statistics type *s,* in year *t.*

Combining short-term and long-term users together leads to the total number of estimated modern users.

**Estimated Modern Users**

$${\mathrm{EMU}}_{s,t=}\sum_{i=1}^{M_{\mathrm{STM}}}U_{m_{{\mathrm{STM}}_{i}},s,t}+\sum_{i=1}^{M_{\mathrm{LTM}}}U_{m_{{\mathrm{LTM}}_{i}},s,t}$$

Where:

*s* is the type of service statistic (commodities to clients, commodities to facilities, visits),

*t* is year,

$U_{m_{{\mathrm{STM}}_{i}},s,t}$ is the number of users of a short-term method, $m_{{\mathrm{STM}}_{i}}$ , of service statistics type *s,* in year *t,*

$U_{m_{{\mathrm{LTM}}_{i}},s,t}$ is the number of users of a long-term method, ${m_{\mathrm{LTM}}}_{i}$ , of service statistics type *s,* in year *t,*

$M_{\mathrm{STM}}$ is the total number of short-term methods,

$M_{\mathrm{LTM}}$ is the total number of long-term methods,

${\mathrm{EMU}}_{s,t}$ is the number of estimated modern users captured by service statistics type *s,* in year *t*

At this point, the EMU calculation represents estimated modern users whose family planning services are recorded in a country’s HMIS. This version of the indicator is used by countries to monitor their programs and track trends in contraceptive use at the national and subnational levels. In most countries, for the EMU to be used as a data source to model contraceptive prevalence, there is one additional adjustment that needs to be made - accounting for family planning services that are not included in a country’s HMIS.

#### Private Sector Adjustment

The three groups - current users, continuing users, and historic users - combine to form an “unadjusted EMU”. It is unadjusted because it does not consider women who may have received contraceptive methods outside of the facilities and/or sectors that report into the national HMIS. The final adjustment integrated into the EMU calculation is intended to make the EMU more representative of the full FP market. In most countries, parts of the private sector report data into HMIS, but it is very rare that the full private sector reports. Efforts have been made to better integrate data from private sector providers, including NGOs providing family planning services and private hospitals and clinics, but few countries have pharmacies or other non-medical sources reporting into the system. This impacts methods differently, for example in many countries, shops are key sources of pills and condoms, and they rarely report into HMIS. The EMU calculation uses a method-specific adjustment factor, applied to the service statistics data, to account for provision in sectors not represented in the available data. This adjustment factor is based on data from DHS (where available) concerning the source of methods for current users of contraception, along with information estimating how much private sector provision is captured in their data.

Multiplying unadjusted EMU (by method) by the method-specific private sector adjustment factors results in the adjusted EMU. All method-specific EMUs are aggregated to form the total EMU. Dividing the adjusted EMU by women of reproductive age gives us the EMU value that can be used in FPET to inform annual family planning estimates.

These transformations are all intended to help the EMU serve as a proxy for contraceptive coverage accounting for the historic provision of LAPMs prior to available data, the continued impact of LAPMs over years following the initial provision, and the provision of services in sectors not represented in HMIS. This positions the EMU to be different, yet complimentary, to CYP, with the EMU being better positioned for active monitoring.

### Operationalizing EMU Calculations at the Country Level

Creating the EMU at the country level requires more than the calculations described above. Unlike publicly available survey datasets which are cleaned and processed, HMIS contain raw data that are entered, aggregated, and managed within country systems which are often under resourced. Additionally, routine service statistics are a living dataset- new data is entered every month and the system may evolve over time in terms of what information is collected. It needs continuous work, which is why it is imperative that a process is embedded in the creation of the EMU to understand the completeness and quality of the data being used.

The Track20 Project created the Excel-based Service Statistics to Estimated Modern Use Tool (SS-to-EMU tool), to walk individuals through the process of producing the EMU. The tool ( *Copyright © 2025 Track20. All Rights Reserved.*), is free to use and available through the Track20 website and GitHub. The same process can be done using R code ( https://doi.org/10.5281/zenodo.14774507), which is available online through Track20’s GitHub repository. The SS-to-EMU Tool performs two functions: it takes the user through a data review and visualization process to easily assess quality and identify trends, facilitating identification and follow-up on data outliers; and calculates the EMU, providing a single metric that can be regularly assessed against other data sources as a measure of progress.

#### SS-to-EMU Data Inputs

A variety of data inputs are used for producing the EMU, see Box 1. Some data are entered by the user, and other data are pre-populated in the tool from trusted data sources.

Box 1. **SS-to-EMU data inputs.**

**Data Required for Calculation of EMU**

▪ Service Statistics data – one or more of the following:
  ▪ FP commodities distributed (to clients or facilities)
  ▪ FP visits
  ▪ FP users
▪ Population data
▪ Public sector market share

**Data Used for Quality Review
**

▪ Reporting coverage rate
▪ FPET & Survey estimates of mCP prevalence & FP method mix

#### Service Statistics

EMU is calculated using any of the four types of family planning routine data typically collected by programs: commodities distributed to clients, commodities distributed to facilities, family planning visits, and family planning users. Service statistics are entered into SS-to-EMU by the user and when possible, users are encouraged to enter more than one type of service statistic to allow for comparison during the data review phase.

The SS-to-EMU Tool makes the previously discussed adjustments to the service statistics entered to convert them to FP Users for the EMU calculation.

#### Population Data

Population estimates for all women ages 15 – 49, by year, are pre-loaded into the SS-to-EMU Tool from the UN Population Division. ^8^ These estimates serve as the denominator for the EMU calculation. Population estimates for all women of reproductive age are preferred as the denominator (as compared to married women of reproductive age) as aggregated service statistics data generally does not contain information about the marital status of the clients who receive FP Services [ 5]. If the user has a preferred data source for population estimates, such as a census, the user may use their own source and replace the pre-populated data.

#### Public Sector Market Share

Data on the distribution of modern users of each method by the source from which they received that method is pulled from DHS to inform the EMU private sector adjustment discussed previously, providing insight on the public sector market share.

#### Reporting Rates

Reporting rates are entered into SS-to-EMU by the user. The reporting rate provides an indication of the share of facilities represented in the HMIS data. Reporting rates are a standard measure in many countries’ HMIS, though they vary extensively in what they represent. Generally, reporting rates refer to the percentage of facilities that submit monthly or annual reporting forms (reporting routine service statistics data) into the national HMIS. Some systems differentiate between “on time” reporting rates (the % of facilities submitting forms by the monthly reporting deadline) and “overall” reporting rates (the % of facilities that have submitted forms at all by the time data is being extracted from the system). Reporting rates recorded in HMIS may not be specific to FP data but can refer to reporting for a broader health area. This depends on whether there is a distinct reporting form for submitting FP-related data or whether FP is included in a broader maternal and reproductive health form or if all health areas are reported up from the facility in a single form. For the purpose of the SS to EMU, FP-specific reporting rates are preferred.

#### Method Prevalence and Method Mix

Data from nationally representative surveys including DHS, MICS, Performance Monitoring for Action (PMA), and other national surveys provide method prevalence and method mix data for benchmarking. SS-to-EMU also uses mCP estimates from FPET for benchmarking.

### Data Quality Review

A comprehensive data quality review is part of the EMU calculation process and embedded in the SS-to-EMU tool. Although anchored in the WHO Data Quality approach, ^9^ Track20 adapted the methodology to center around FP and streamlined it into a largely automated process. The result is a comprehensive but easy-to-follow process that generates data visualizations to help users easily identify data anomalies. The standardized process is a first for family planning data and a critical step for improved data management and use.

#### Assessing Data Completeness

Because routine service statistics are based on facilities reporting into HMIS, data completeness, an important element of data quality, is dependent on high reporting rates. A reporting rate of 100% would indicate that all services delivered by the public sector are captured in the HMIS data. The Track20 project typically recommends a reporting rate of 80% or higher for service statistics to be included in the EMU trend to ensure data are representative and largely complete. The SS-to-EMU Tool does not adjust for low reporting rates as it does for missing private sector data. Instead, low reporting rates will result in the exclusion of EMUs in that year from trend data. However, no matter the reporting rate, working through the data review and assessment processes can be an important and routine step incorporated into a country’s family planning monitoring system.

#### Internal Benchmarking

Users are encouraged to enter as many types of service statistics into the SS-to-EMU tool as are available in their system. This allows the user to examine the relationship between different types of service statistics, which can provide important internal benchmarking. The SS-to-EMU Tool displays the inputted data in ways that allow the user to easily identify outlier data points, facilitating follow-up and potential data corrections, if necessary.

Ideally, trends in the different data types should be similar, as should the distribution of methods. There are some typical relationships between data sources that users can look for. For example, LMIS data on commodities sent to facilities should typically be higher than data on commodities given to clients because not all methods sent to facilities are used. Commodities to clients and visits data for single dose methods (IUDs, DMPA-IM, implants) will be similar because one commodity unit is distributed per visit. Sterilization is more commonly recorded in visits and users, but less likely to be reported in commodities data, resulting in a potential gap when only looking at one type of data.

The tool helps walk through these different relationships and uses clear visualizations that can help users see how their data relate to each other and if the relationships make sense.

#### External Benchmarking

The SS-to-EMU tool is unique in that it includes routine service statistics alongside survey data and mCP from FPET global projections, so tool users can benchmark their EMU trends (derived from their service statistics) against trusted external data.

For years with survey data available, users can compare rates and absolute numbers. For absolute numbers, estimated users by method can be compared to the number of users calculated from survey method prevalence multiplied by the population of women of reproductive age. Although one would not expect estimated users from surveys and service statistics to be equal (due to methodology and adjustments), how they compare can provide some indication of whether the data is generally consistent with external sources or can point to potential issues with the data inputs. For example, one would expect that unadjusted estimated users (before one accounts for the private sector) would generally be lower than survey-based estimates of users and that the private sector adjustment would bring the estimate of users closer to the survey-based estimates. There is a specific graph included in the data review that shows the impact of the private sector adjustment factor in comparison to survey-based estimates and allows the user to revisit the adjustment factors if the impact of those adjustments appears to over- or under-estimate use. If the estimates of users are substantially higher than survey-based estimates, especially for a specific method, that may point to potential issues with double counting or quality issues in HMIS. Finally, one would also expect that estimated users is likely to diverge from survey data the farther out one is from a survey. When comparing service statistics from 2023 against a survey conducted in 2018, one would expect some variation, especially as program priorities and the scale of method use may have changed.

While it is expected that EMU and surveys will trend together, there are some specific FP methods that can cause particular challenges for estimating use, generally related to how data is recorded and gaps between distribution and consumption. LAM (Lactation Amenorrhea Method) is not always recorded in the HMIS, or if it is recorded, it is in reference to LAM counseling as opposed to measured use. The tool allows for the exclusion of condoms because of the various motivations for condom use and in accurate recording in HMIS. Overall, understanding how and why different methods may result in an EMU that varies from survey data can help the user identify potential data quality issues, make decisions about how to use the data, and determine which data type is providing the most accurate picture of use.

In addition to doing comparisons in the year of a survey, users also look at annual rates of growth between surveys and the corresponding trends to EMU. Major differences in rates of change can highlight potential data quality issues.

### Decision to Use EMU for Family Planning Monitoring

After completing the data review process, the SS-to-EMU tool guides the user through a final review of how each of the EMUs derived from the different types of service statistic entered performed during the quality review.

For countries where routine data quality is poor, the graphics produced by the data quality review provide a useful tool for advocating for improvement in data quality. For countries where data quality is sufficient, the EMU indicator can be used directly to monitor change and as an input in FPET, thus influencing the estimation of mCP.

The number of countries whose service statistics were of high enough quality to use their EMU in their FPET estimates reported in the FP2020/FP2030 Annual Progress Report has grown from 5 countries in 2015, to as high as 20 in a single year. Because HMIS data and systems are dynamic and issues arise, a country may not be able to use EMU every year.

## Results

### EMU Use in Family Planning Program Monitoring

#### Global Level

Aligned with its original intent, the EMU indicator has supported annual progress reporting for the FP2020 initiative, and its follow-on FP2030, as well as the Ouagadougou Partnership, through its use as an input into FPET and the resulting estimates of key family planning indicators (mCP, Unmet Need, and Demand Satisfied). EMU allows for the introduction of service statistics into family planning modeling, particularly beneficial in years without surveys. It provides better representation of recent changes in family planning programs, impacting estimates and narrowing confidence intervals. Since 2014, 26 countries have used EMU to inform their annual FPET estimates in at least one year of reporting.

Because EMU is a standard measure, it has been adopted by various donors and partners as part of their annual monitoring process. EMU is a required core indicator on the USAID MOMENTUM suite of grants which implements activities across 38 countries. EMU has also been adopted by the Countdown to 2030 global movement for women’s, children’s, and adolescents’ health as its approach to measure coverage for family planning across 81 focus countries.

In addition, because the EMU can be calculated at sub-national levels, it can be particularly useful for tracking progress in specific sub-national geographies where different investments are made and would provide governments, projects, and donors with a common indicator to use across national and subnational investments.

#### Country Level

The SS-to-EMU tool is used to calculate EMU independently in over 30 countries as part of routine annual family planning monitoring and EMU is now auto-calculated monthly within 13 [ 6] country FP HMIS environments through Track20’s FP DataPro. FP DataPro is an FP and MNH environment available for download from the DHIS2 App Hub that includes EMU calculation and aspects of the SS-to-EMU data quality review, along with complex data analytics and visualizations to support active monitoring. ^10^

Regular utilization of the data quality review through the SS-to-EMU tool facilitates the identification of outliers and other data anomalies and supports quick follow-up with facilities to confirm entries and make corrections if necessary, improving data quality over time.

Because EMU is a service-based indicator and can be calculated annually, quarterly, or monthly, it is responsive to immediate changes in service volume. This means that the EMU will reflect trend shifts due to program scale-ups, service disruptions, and other changes long before a survey would, providing more immediate feedback. EMU proved especially helpful during the COVID-19 pandemic as countries strove to understand whether COVID-19 impacted FP uptake and use. Subnational EMU calculations provide additional depth. This has allowed countries to make more responsive and targeted programmatic changes between surveys.

## Discussion

Creating a standardized routine service statistic based indicator has provided an opportunity to look at data across countries and provided countries with a new option for national and subnational monitoring. As an indicator based on HMIS, not surveys, understanding how to interpret and use the EMU is important. Although the EMU provides an estimate of total contraceptive use, it should not be interpreted as being the same as mCP produced from a probabilistic survey. As described above, HMIS data in most countries does not contain data representing the total market of family planning services. There are channels of delivery, such as pharmacies and markets, that are rarely captured in HMIS. Although the process of producing the EMU includes an adjustment for this missing data, the adjustment factors are from the most recent survey and may be outdated. Due to this, when comparing the EMU to mCP, the expectation is that the levels may not match, but the trends should. The assumption is that changes in the percentage of women that use services from channels captured in HMIS and those that are not happen slowly over time. As the trend of incorporating more data into HMIS continues, this adjustment will have a smaller and smaller impact. To mitigate this risk, uncertainty has been added to the private sector adjustment when the EMU is added into FPET to produce mCP.

Since the EMU has been used across multiple countries for over a decade, Track20 has been able to take feedback and lessons learned from country stakeholders and refine both the indicator and the process used for its production. This has led to improvements in how the indicator is calculated. For example, there have been changes made about how the historical users are calculated which are seen in the differences if a method is labeled as being scaled up or already at scale. A current area of exploration is the global continuation rates created for CYP factors that are used for long-acting methods. As explained, the current approach is to use the global factors in the creation of the EMU. Although using country specific continuation rates would be ideal, a lack of data availability prevents this approach. Using regional continuation rates has already been explored, but did not provide an improvement because the variations seen within regions is the same as what is seen globally. There are two areas currently being explored. The first one is a simple approach that allows the user to adjust the global continuation curve up or down based on available country data. This would not change the shape of the curve, just the level at where it begins. In countries with very high discontinuation this could adjust the EMU to be more aligned with actual use. The second is to make changes to the implant curve. Looking at recent DHS data shows that continuation of IUDs is a close match to the CYP global continuation curve. However, a first look at implant data suggests there are some differences. The CYP curve was created using Norplant data because at the time there was very little data about usage of the new generation of implants. Newer DHS data shows that there may be a divergence in continuation with the new implants in the final year of effectiveness. Conceptually this makes sense because implants now, which are of shorter duration than the 7 year Norplant, are more likely to be used as a spacing method. One of the larger discrepancies seen between use in service statistics and use in surveys is for implants. Use in HMIS is often higher than use in surveys. Changes to the continuation curve used to calculate the EMU would help to mitigate these differences and could better reflect actual use.

Although there are limitations in terms of the representativeness HMIS and the quality of routine service statistic data, the impulse to have a proportional indicator is seen everywhere. Most countries already have a process to do this, but it is often oversimplified and ignores data quality. The EMU is the most comprehensive effort yet to standardize the calculation within existing metrics and systems. It is adaptable to data coming from multiple systems of data collection and reporting, allowing most countries to produce the indicator.

## Conclusions

EMU is a new routine service statistics-based family planning indicator that contributes to more accurate and timely monitoring of family planning programs. EMU can help countries more closely monitor their own progress toward family planning goals and advances the FP monitoring field by facilitating cross-country comparisons and a standardized measure to track trends. Subnational EMUs provide important information to guide targeted programming and fills gaps where survey data is unavailable. As an input into FPET, EMU can increase the confidence of annual estimates of mCP, unmet need, and demand satisfied by modern methods and provide early indications of changes in trends in family planning use. The process of data review incorporated in the SS-to-EMU Tool has been shown to improve the quality, management, and use of family planning data for decision making. Since its introduction, EMU has been included in the routine list of indicators collected and analyzed by countries, donors and partners, standardizing how FP progress is tracked and discussed.

## Data Availability

This paper is theoretical, all data in this paper is for demonstration purposes only and does not reflect any individual or country. The SS-to-EMU Excel Tool s publicly available on the Track20 website, free of charge: https://www.track20.org/pages/track20_tools/SS_to_EMU_tool.php Supplementary material: The R code used to calculate EMU is available at the following GitHub repository: https://github.com/kristinbietsch/SStoEMU-National-and-Subnational MIT LicenseCopyright (c) 2025 Kristin Bietsch Archival source code is available from https://doi.org/10.5281/zenodo.14811417 Data are available under the terms of the Creative Commons Attribution 4.0 International license (CC-BY 4.0). Permission is hereby granted, free of charge, to any person obtaining a copy of this software and associated documentation files (the “Software”), to deal in the Software without restriction, including without limitation the rights to use, copy, modify, merge, publish, distribute, sublicense, and/or sell copies of the Software, and to permit persons to whom the Software is furnished to do so, subject to the following conditions: The above copyright notice and this permission notice shall be included in all copies or substantial portions of the Software. The software is provided “as is”, without warranty of any kind, express or Implied, including but not limited to the warranties of merchantability, Fitness for a particular purpose and noninfringement. In no event shall the Authors or copyright holders be liable for any claim, damages or other Liability, whether in an action of contract, tort or otherwise, arising from, Out of or in connection with the software or the use or other dealings in the Software.

## Footnotes

^1^

https://www.track20.org/

^2^

https://dhsprogram.com/

^3^

https://mics.unicef.org/

^4^
In Bangladesh, India, and Nepal the CYP factor for female sterilization is 13 years, as opposed to the global 10, to account for their earlier age at sterilization.

^5^
In a few cases, specifically countries where FP services are only offered to married women or it can generally be assumed that unmarried women are not accessing FP through the public sector, population estimates for married women have been used as an alternative denominator.

^6^
The automated EMU calculation is through FP DataPro in 12 countries and the predecessor version, the DHIS2 FP Module, in 1 country.
